# Development and validation of a metastasis-associated prognostic signature based on single-cell RNA-seq in clear cell renal cell carcinoma

**DOI:** 10.18632/aging.102434

**Published:** 2019-11-20

**Authors:** Chuanjie Zhang, Hongchao He, Xin Hu, Ao Liu, Da Huang, Yang Xu, Lu Chen, Danfeng Xu

**Affiliations:** 1 Department of Urology, Ruijin Hospital, School of Medicine, Shanghai Jiaotong University, Shanghai, China; 2 First Clinical Medical College of Nanjing Medical University, Nanjing, China

**Keywords:** single-cell RNA-seq, metastasis-associated genes, progression, tumor mutation burden, TCGA

## Abstract

Single-cell RNA sequencing (scRNA-seq) was recently adopted for deciphering intratumoral heterogeneity across cell sub-populations, including clear cell renal cell carcinoma (ccRCC). Here, we characterized the single-cell expression profiling of 121 cell samples and found 44 metastasis-associated marker genes. Accordingly, we trained and validated 17 pivotal metastasis-associated genes (MAGs) in 626 patients incorporating internal and external cohorts to evaluate the model for predicting overall survival (OS) and progression-free survival (PFS). Correlation analysis revealed that the MAGs correlated significantly with several risk clinical characteristics. Moreover, we conducted Cox regression analysis integrating these independent clinical variables into a MAGs nomogram with superior accuracy in predicting progression events. We further revealed the differential landscape of somatic tumor mutation burden (TMB) between two nomogram-score groups and observed that TMB was also a prognostic biomarker; patients with high MAGs-nomogram scores suffered from a higher TMB, potentially leading to worse prognosis. Last, higher MAGs-nomogram scores correlated with the upregulation of oxidative phosphorylation, the Wnt signaling pathway, and MAPK signaling crosstalk in ccRCC. Overall, we constructed the robust MAGs through scRNA-seq and validated the model in a large patient population, which was valuable for prognostic stratification and providing potential targets against metastatic ccRCC.

## INTRODUCTION

Kidney cancer is a common malignancy of the urinary system mostly originating from the renal tubular epithelium, and its incidence rate has increased worldwide in recent years. The number of newly diagnosed cases in the USA has grown up to 65,000 per year, leading to approximately 15,000 deaths annually according to the recent cancer statistic report [1]. Clear cell renal cell carcinoma (ccRCC), the most common histopathological type of sporadic kidney cancer (~80%), was demonstrated to be associated with worse survival outcomes compared with other subtypes of tumors, including papillary renal cell carcinoma, chromophobe renal cell carcinoma and collecting duct carcinoma [2]. Nearly 20% of ccRCC cases progressed to advanced stages at the onset of diagnosis, and the 5-year overall survival (OS) rate of metastatic cases decreased to approximately 10% [3]. With the development of surgical intervention, radiotherapy and immunotherapies, combination strategies have been largely optimized for tumor management. However, the actual clinical efficiency remained marginally improved, and 30% of localized ccRCC patients inevitably suffered from recurrence and cancer-related progression [4]. Though various signaling crosstalk pathways involved in carcinogenesis have been proposed as underlying treatment targets consisting of mammalian target of rapamycin (mTOR), vascular endothelial growth factor (VEGF) or mitogen-activated protein kinase (MAPK), drug resistance and limited progression-free survival (PFS) still exist, especially for metastatic ccRCC [5–7]. Therefore, investigations on the molecular mechanisms underlying the metastasis or progression of ccRCC and new novel targets are urgently needed.

Intensive studies have been conducted to identify numerous biomarkers associated with the survival of ccRCC for predicting prognosis, including mutated drivers, cancer-related noncoding RNA, risk methylated loci, and immune signatures in the tumor microenvironment [8–10]. However, metastasis and tumor recurrence are relatively more essential determinants not only for the selection of treatment strategies but also for the overall prognosis of patients. Previous researchers have already attempted to investigate several pivotal biomarker associated with metastasis from bulk transcriptome profiles [11, 12]. The screening and identification of valuable metastasis-related genes could expand our comprehensive understanding of the differential genomic alterations between primary and metastatic ccRCC. Moreover, these hazard biomarkers could provide more options for the optimization of strategies or for the effective prediction of progressive events.

Recent advances in single-cell RNA sequencing (scRNA-seq) have facilitated the transcriptional classification of cell types in many malignancies, including pancreatic ductal adenocarcinoma (PDAC), breast cancer and lung cancer [13, 14]. Furthermore, scRNA-seq has been expected to possess clinical utility in cases of refractory cancers and is a noninvasive method for monitoring circulating cancer cells, analyzing intratumor heterogeneity and estimating recurrent tumors with sensitivity [15]. Chong Li et al. successfully utilized single-cell exome sequencing and found that KCP, LOC440040, and LOC440563 mutations are novel renal cancer stem cell drivers [16]. Accordingly, we investigated significant marker genes among subpopulations of primary and metastatic ccRCC cells from single-cell expression profiling [17].

In this study, we derived and characterized the genomic features and marker genes between primary and metastatic tumors using scRNA-seq profiling from high-quality tumor cells isolated from parental metastatic renal cell carcinoma (mRCC), patient-derived xenografts of metastatic renal cell carcinoma (PDX-mRCC) and patient-derived xenografts of primary renal cell carcinoma (PDX-pRCC). In addition, we further obtained the transcriptome data, somatic mutation variation data and clinical data of 628 patients from The Cancer Genome Atlas (TCGA) and the International Cancer Genome Consortium (ICGC) database. We conducted a large-sample and multiomics analysis of metastasis-associated genes (MAGs) to validate the robustness of the signature in predicting the progression of ccRCC, which could shed light on further individualized treatment.

## RESULTS

### Single-cell RNA-seq profiling and screening of metastasis-associated marker genes

**Figure 1:**
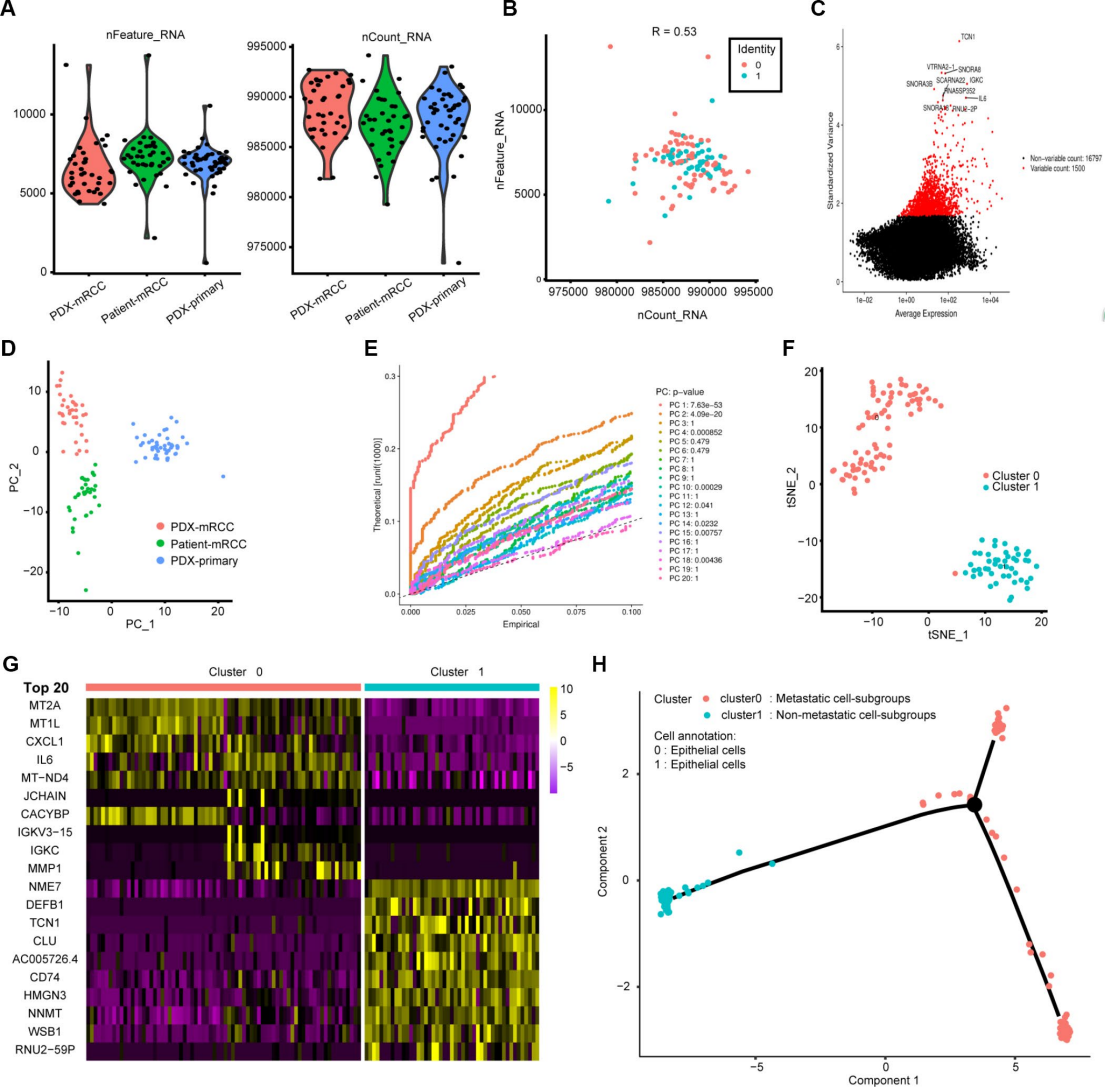
**Characterization of single-cell RNA sequencing from 121 cells and screening of marker genes.** (**A**, **B**) Quality control of scRNA-seq for three cell sub-populations. We filtered out the cells with poor quality and analyzed the positive associations between detected gene counts and sequencing depth. (**C**) we identified the gene symbols with significant difference across cells and drawn the characteristic variance diagram. (**D**, **E**) The principal component analysis (PCA), a linear dimensionality reduction method, was ultilized to identify the significantly available dimensions of data sets with estimated P value. Accordingly, we classified the cell groups into three categories. (**F**) Based on available significant components from PCA, we conducted another nonlinear dimensionality reduction, TSNE algorithm, to successfully divided the cells into two clusters, in accordance with actual cell types. (**G**) Differential analysis with logFC =0.5 and adjPval =0.05 was constructed between two clusters to identify significant marker genes and we exhibited the top 20 in heatmap package. (**H**) Cell annotations and trajectory analysis revealed the tendency curve from primary RCC to metastatic ones, indicating the genomic alternations between them.

We acquired 121 cell samples with superior quality isolated from three subpopulations consisting of patient-derived mRCC, PDX-mRCC and PDX-pRCC (Table 1). We combined the sequencing data of 121 files into one matrix and transformed the gene symbols based on the human GTF file. The quality control chart is shown in Figure 1A, where the range of detected gene numbers and the sequencing count of each cell are illustrated. We accordingly excluded cells with a percentage of mitochondrial sequencing count > 5%. Additionally, we observed a significantly positive correlation between the detected gene numbers and the sequencing depth with Pearson's r = 0.53, as shown in Figure 1B. The variance analysis revealed the top 10 significantly differentially expressed genes across the cell samples, including TCN1, IL-6, RNU2-2P, IGKC and SNORA1B (Figure 1C). Furthermore, we used the principal component analysis (PCA) method and screened the significantly correlated genes in each component. The top 30 significantly correlated genes are shown via heatmap and dot plot in Supplementary Figure 1. In addition, we mapped the cells into two dimensions based on the PC_1 and PC_2 components, and the three correct independent cell subpopulations indicated the preferable clustering efficiency during the PCA procedure (Figure 1D). The other components were calculated with an estimated *P* value, and we selected the significant components for subsequent analysis. Apart from utilizing the linear dimensionality reduction method, we also used the t-Distributed Stochastic Neighbor Embedding (t-SNE) algorithm, commonly adopted for the visualization of high dimensional data, to further precisely cluster the populations of cells, in which we successfully classified the samples into two subgroups consisting of primary and metastatic cells (Figure 1F, Supplementary Table 1). Accordingly, we performed differential analysis using the limma package and identified a total of 265 marker genes with | log fold change (FC) | > 0.5 and adjPval < 0.05 (Supplementary Table 2). We selected 44 genes with | logFC | > 1 as the hub MAGs. The top 20 differential genes between the two clusters in the heatmap plot are illustrated in Figure 1G. Additionally, we annotated the evaluated cell type for each cell sample using the marker genes (Supplementary Table 3) and characterized the integrative trajectory of the single-cell sequencing results. Though all the cells in the two clusters were annotated as epithelial cells, we observed a significant tendency curve from cluster 1 of the primary cells to cluster 0 of the metastatic cells, indicating the underlying transcriptional heterogeneity between two tumor subpopulations in ccRCC (Figure 1H).

**Table 1:** Tumor cells from the parental mRCC, PDX-mRCC and PDX-primary in GSE73121 were finally analyzed in this study after filtering out poor quality cells.

**Category**	**Cell count**	**Percentage (%)**
PDX-primary	48	39.67
PDX-mRCC	37	30.58
Patient-mRCC	36	29.75
Total	121	100

### Validation of MAGs in internal and external ccRCC populations

**Figure 2:**
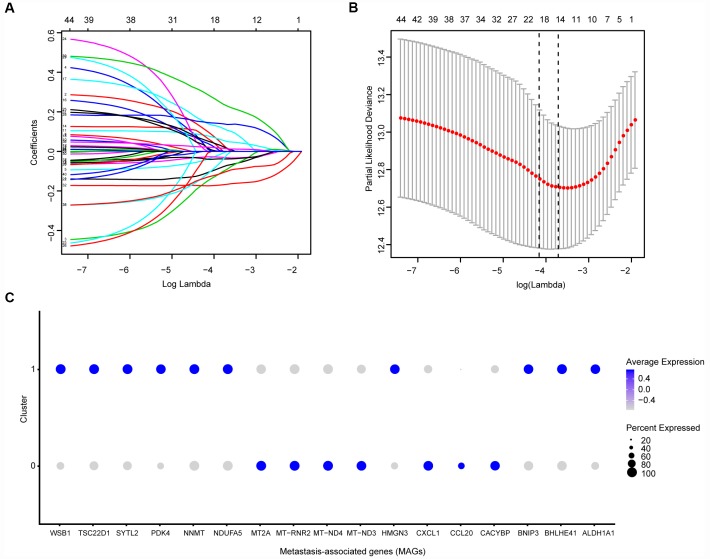
**Identification of prognostic metastasis associated genes.** (**A**, **B**) We conducted the LASSO method based on glmnet package and identified the 17 prognostic genes in TCGA training cohort, where the optimal cutoff value was -4 and the minimum account of genes was 17. © Meanwhile, we also illustrated the significantly differential expressions of 17 prognostic genes in two clusters via bubble plot.

**Figure 3:**
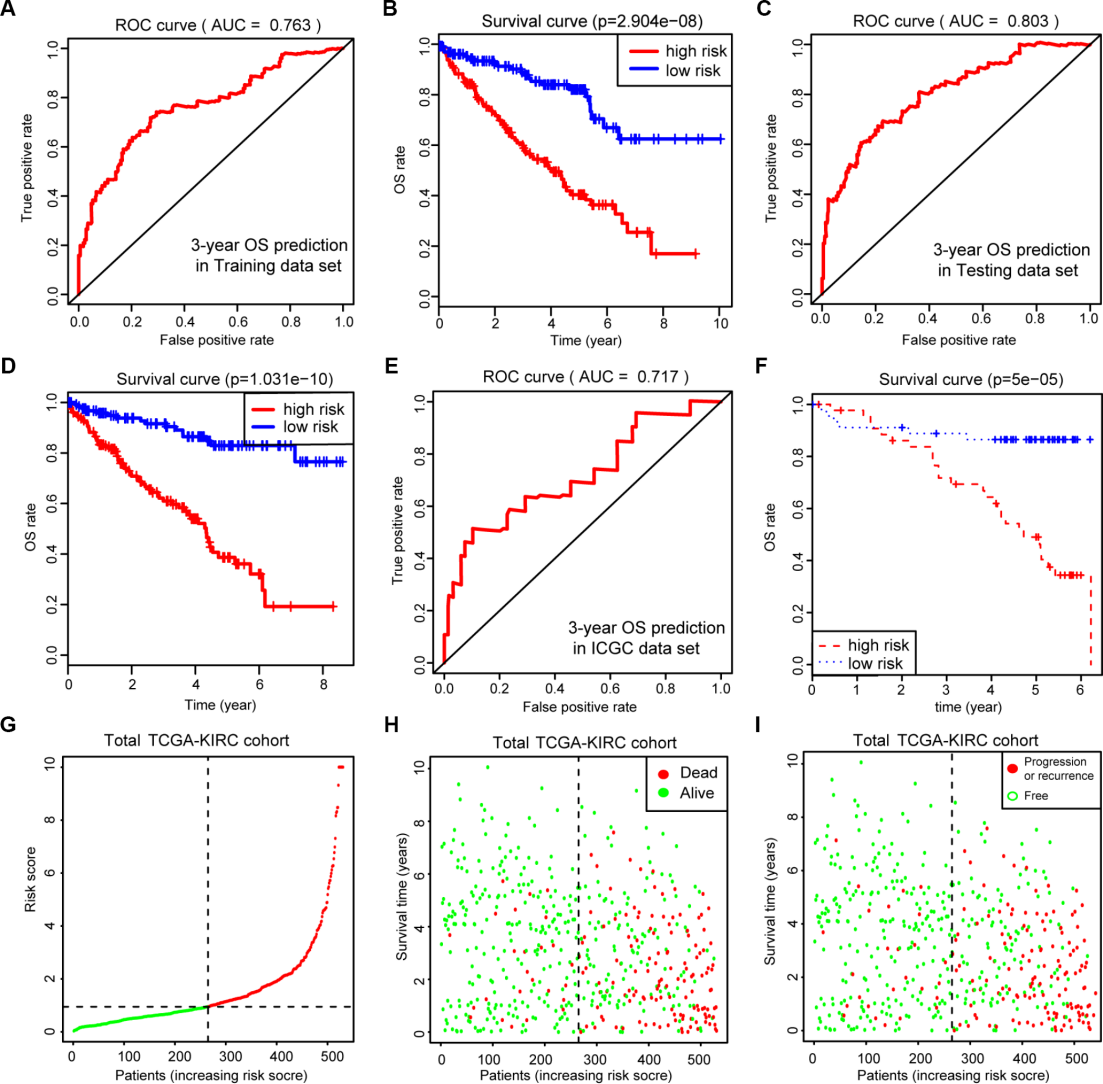
**Internal and external validation of MAGs to determine its clinical predictive value.** (**A**, **C**) The AUCs of ROC curves were 0.763 and 0.803 in predicting 3-year OS events in training and testing cohorts, respectively. (**B**, **D**) Besides, Kaplan-Meier analysis indicated that patients with high MAGs-score suffered significantly worse OS outcomes (*P* = 2.904e-08), which was validated consistently in testing cohort with *P* = 1.031e-10. (**E**, **F**) In addition, we also proved our findings in an independent ICGC cohort and observed the similar statistical results. (**G**–**I**) We further integrated MAGs signature with survival analysis in the total TCGA-KIRC cohort and distribution plots suggested that high MAGs risk scores correlated with more dead and recurrence/progression cases.

Before conducting the Cox analysis, we first adopted the merge function in R studio to integrate the expression profiles of the 44 differential hub MAGs with corresponding survival information in the total TCGA-Kidney Renal Clear Cell Carcinoma (KIRC) data set. We used the least absolute shrinkage and selection operator (LASSO) method and identified 17 prognostic genes in the training cohort (Figure 2A and 2B). The complete clinical information of the ccRCC patients included in our study is shown in Table 2. Additionally, we illustrated the significant differential expression of 17 prognostic genes in two clusters (Figure 2C and Supplementary Figure 2). The MAG signature was then established based on multivariate Cox regression, and the areas under the curve (AUCs) of the receiver operating characteristic (ROC) curves were 0.763 and 0.803 for predicting 3-year OS events in the training and testing cohorts, respectively (Figure 3A and 3C). In addition, Kaplan-Meier analysis indicated that patients with high MAG scores suffered significantly worse OS outcomes (*P* = 2.904e-08), which was validated consistently in the testing cohort with *P* = 1.031e-10. (Figure 3B and 3D). In addition, we also demonstrated our findings in an independent ICGC cohort and observed similar statistical results (Figure 3E and 3F, Supplementary Table 4). Overall, we further integrated the MAG signature with survival analysis in the total TCGA-KIRC cohort, and distribution plots suggested that high MAG risk scores correlated with more cases of death or recurrence/ progression (Figure 3G, 3H and 3I). The Cox regression results and Kaplan-Meier analysis of the 17 hub genes in the TCGA-KIRC cohort are shown in Table 3 and Supplementary Figure 3.

**Table 2:** Clinical characteristics of total 628 ccRCC patients included in this study.

**Variables**	**Total TCGA-KIRC**	**Training group**	**Testing group**	**ICGC cohort**
	**(N = 537)**	**(N = 265)**	**(N = 265)**	**(N = 91)**
**Age (Mean ± SD)**	60.59 ± 12.14	60.21 ± 12.18	59.92 ± 12.04	60.47 ± 9.97
**Follow-up (y)**	3.12 ± 2.23	3.17 ± 2.26	3.06 ± 2.21	4.14 ± 1.73
**Status**				
Alive	367 (68.34)	175(66.04)	189(71.32)	61 (67.03)
Dead	170 (31.66)	90(33.96)	76(28.68)	30 (32.97)
**Gender**				
Male	346 (64.43)	172(64.91)	172(64.91)	52 (57.14)
Female	191 (35.57)	93(35.09)	93(35.09)	39 (42.86)
**AJCC-T**				
T1	275 (51.21)	144(54.34)	127(47.92)	54 (59.34)
T2	69 (12.85)	30(11.32)	39(14.72)	13 (14.28)
T3	182 (33.89)	83(31.32)	96(36.23)	22 (24.18)
T4	11 (2.05)	8(3.02)	3(1.13)	2 (2.20)
**AJCC-N**				
N0	240 (44.69)	116(43.77)	123(46.41)	79 (86.81)
N1	17 (3.17)	4(1.51)	12(4.53)	2 (2.20)
Unknow	280 (52.14)	145(54.72)	130(49.06)	10 (10.99)
**AJCC-M**				
M0	426 (79.33)	207(78.11)	213(80.38)	81 (89.01)
M1	79 (14.71)	42(15.85)	36(13.58)	9 (9.89)
Unknow	32 (5.96)	16(6.04)	16(6.04)	1 (1.10)
**Pathological stage**				
I	269 (50.09)	142(53.58)	123(46.42)	-
II	57 (10.61)	27(10.19)	30(11.32)	-
III	125 (23.28)	51(19.25)	72(27.17)	-
IV	83 (15.46)	44(16.60)	38(14.34)	-
Unknow	3 (0.56)	1(0.38)	2(0.75)	-
**Grade**				
G1	14 (2.61)	4(1.51)	10(3.77)	-
G2	230 (42.83)	122(46.04)	105(39.62)	-
G3	207 (38.54)	102(38.49)	104(39.25)	-
G4	78(14.53)	34(12.83)	41(15.47)	-
Unknow	8(1.49)	3(1.13)	5(1.89)	--
**MAGs levels**				
High	265(49.35)	132(49.81)	132(49.81)	45(49.45)
Low	265(49.35)	133(50.19)	133(50.19)	46(50.55)
Unknown	7(1.30)	-	-	-

Data are shown as n (%).

Abbreviations: TCGA, The Cancer Genome Atlas; ICGC, International Cancer Genome Consortium; AJCC, American Joint Committee on Cancer.

**Table 3:** Identification of 17 prognostic MAGs related with survival and progression in total TCGA-KIRC cohort.

**Gene symbol**	**Description**	**OS (*P* value)**		**PFS (*P* value)**	
		**Univariate Cox**	**Multivariate Cox**	**Univariate Cox**	**Multivariate Cox**
ALDH1A1	aldehyde dehydrogenase 1 family member A1	0.000	0.012	0.001	0.028
BHLHE41	basic helix-loop-helix family member e41	0.092	0.005	0.085	0.004
BNIP3	BCL2 interacting protein 3	0.000	0.000	0.002	0.001
CACYBP	calcyclin binding protein	0.001	0.069	0.874	0.009
CCL20	C-C motif chemokine ligand 20	0.035	0.013	0.077	0.075
CXCL1	C-X-C motif chemokine ligand 1	0.000	0.005	0.000	0.045
HMGN3	high mobility group nucleosomal binding domain 3	0.004	0.012	0.000	0.001
MT-ND3	mitochondrially encoded NADH dehydrogenase 3	0.015	0.007	0.082	0.007
MT-ND4	mitochondrially encoded NADH dehydrogenase 4	0.004	0.001	0.006	0.000
MT-RNR2	mitochondrially encoded 16S RNA	0.053	0.003	0.831	0.001
MT2A	metallothionein 2A	0.000	0.012	0.000	0.003
NDUFA5	NADH:ubiquinone oxidoreductase subunit A5	0.002	0.010	0.026	0.023
NNMT	nicotinamide N-methyltransferase	0.007	0.020	0.001	0.120
PDK4	pyruvate dehydrogenase kinase 4	0.000	0.000	0.000	0.020
SYTL2	synaptotagmin like 2	0.062	0.001	0.023	0.063
TSC22D1	TSC22 domain family member 1	0.000	0.043	0.000	0.067
WSB1	WD repeat and SOCS box containing 1	0.000	0.000	0.008	0.017

### Correlation analysis of MAGs with clinical characteristics

**Figure 4:**
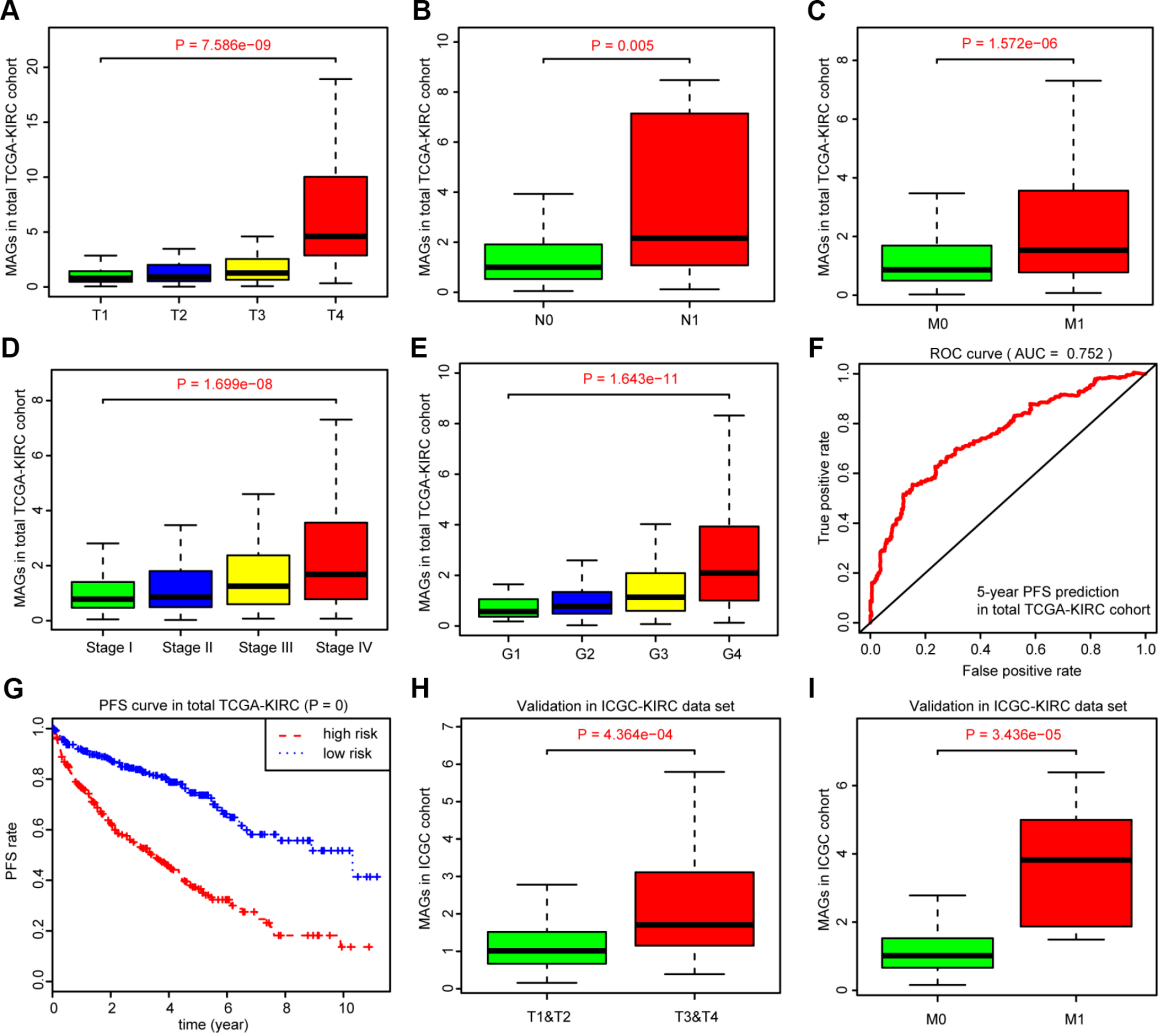
**Correlation analysis between MAGs with other clinical variables and predictive efficiency of MAGs in PFS.** (**A**–**E**) Kruskal-Wallis test revealed that increasing MAGs-score correlated with higher T stages (*P* = 7.586e-09), higher positive rate of lymph nodes (*P* = 0.005), advanced metastatic stages (*P* = 1.572e-06), poor pathological stages (*P* = 1.699e-08) and progressive tumor grades (*P* = 1.643e-11). (**F**, **G**) Moreover, the MAGs signature possessed superior significance in 5-year PFS prediction with AUC = 0.752 in total TCGA-KIRC cohort and patients with high MAGs-score suffered more hazards in tumor recurrence or progression with log-rank test of *P* = 0. (**H**, **I**) Correlation analysis of MAGs with T, M stages in ICGC validation cohort.

Given the clinical significance of MAGs in ccRCC, we sought to investigate the potential relationships among the MAGs with other clinical features. The Kruskal-Wallis test revealed that increasing MAG scores correlated with higher T stages (*P* = 7.586e-09), higher positive rates of lymph nodes (*P* = 0.005), advanced metastatic stages (*P* = 1.572e-06), poor pathological stages (*P* = 1.699e-08) and progressive tumor grades (*P* = 1.643e-11). Moreover, the MAG signature possessed superior significance in predicting 5-year PFS with an AUC of 0.752 in the total TCGA-KIRC cohort (Figure 4F), and patients with high MAG scores were proven to have greater hazards regarding tumor recurrence or progression with a log-rank test *P* = 0 (Figure 4G). Furthermore, we validated the underlying relationships in another ICGC data set, in which we found that MAG scores remained significantly associated with T stage (*P* = 4.364e-04) and metastatic status (*P* = 3.436e-05).

### Construction of the MAG nomogram for predicting progression

**Figure 5:**
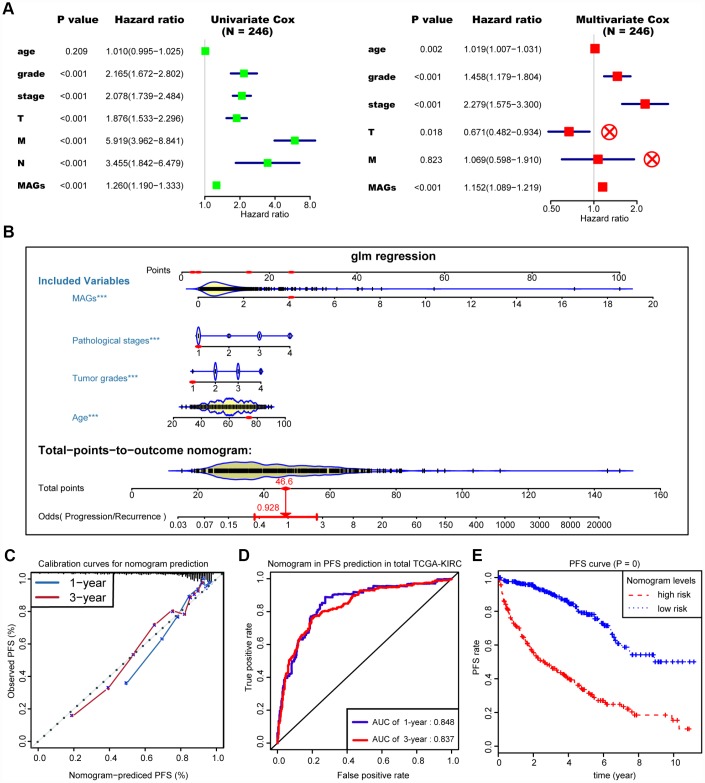
**Construction and assessment of MAGs-nomogram for predicting progression.** (**A**) Univariate- and multivariate Cox regression analysis for screening appropriate and significant features into final nomogram model. (**B**) Ultilizing the glm regression algorithm, the MAGs-nomogram incorporating these four variables was developed and the TCGA-KIRC cohort was classified into high and low groups according to the median of MAGs-nomogram scores. (**C**) Calibration curve was drawn to depict the well curve fitting between predicted 1-year or 3-year progression events and actual observed outcomes. (**D**, **E**) Meanwhile, the AUCs of MAGs-nomogram in predicting 1-year and 3-year progression outcomes were up to 0.848 and 0.837, respectively. Survival analysis also suggested that the MAGs-nomogram was determined to be a significant predictor in PFS of ccRCC with *P* = 0.

We then integrated the MAG signature with other independent clinical variables to construct a comprehensive model for monitoring progression in ccRCC. We excluded the N stage factor for more than half of the missing cases and disregarded the variables with no statistical significance in the multivariate Cox regression model. We finally selected four independent risk features into our model consisting of age, tumor grade, pathological stage and MAG signature (Figure 5A). Utilizing the generalized linear model (GLM) regression algorithm, the MAG nomogram incorporating these four features was developed and is shown in Figure 5B. We classified the TCGA-KIRC cohort into high and low groups according to the median of the MAG nomogram scores. A calibration curve was drawn to depict the fitted model in terms of the agreement between the predicted 1-year or 3-year progression/recurrence events and the actual observed outcomes (Figure 5C). The AUCs of the MAG nomogram in predicting 1-year and 3-year progression outcomes reached up to 0.848 and 0.837, respectively (Figure 5D). Survival analysis also suggested that the MAG nomogram was a significant predictor of ccRCC PFS with *P* = 0 (Figure 5E).

### Differential somatic mutation burden landscape between two nomogram-score levels

**Figure 6:**
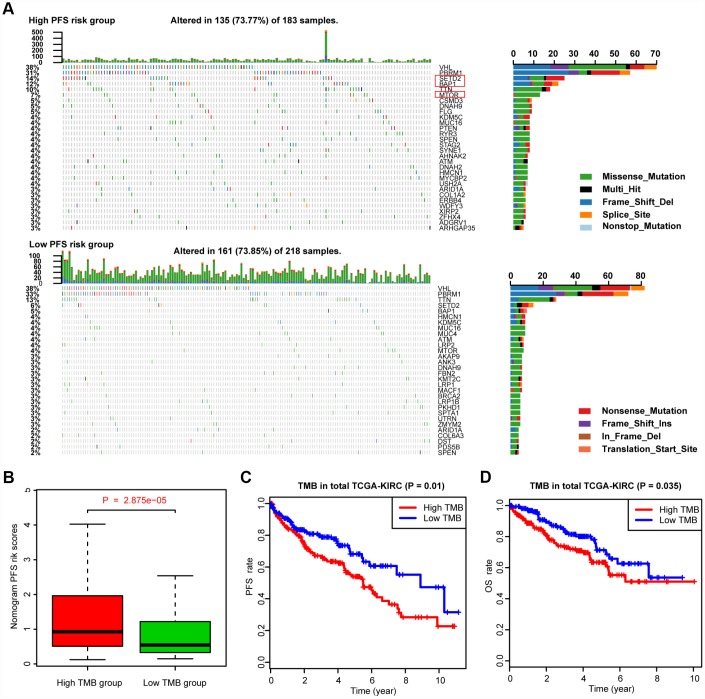
**Differential landscape of somatic mutation burden between high and low MAGs-nomogram levels.** (**A**) The mutational landscape reflected that mutated events occurred more frequently in high Nomogram-score group than that in low group. Besides, the Chi-square test revealed that VHL, PBRM1, SETD2 and BAP1 especially harbored more mutants compared with that in low risk group. (**B**) Wilcoxon rank-sum test suggested that the MAGs-nomogram risk scores were significantly higher in high TMB group than that in low TMB group (*P* = 2.875e-05). (**C**, **D**) Additionally, we found that higher TMB levels were associated with more risks of progression events with *P* = 0.01 and worse OS outcomes with *P* = 0.035.

We defined and calculated the TMB variable in the TCGA-KIRC cohort, matched with corresponding MAG nomogram scores (Supplementary Table 5). The mutational landscape indicated that mutation events occurred more frequently in the high nomogram-score group than in the low group. In addition, we calculated the differential mutation rate of mutants distributed in more than 5% of the samples, and the chi-square test revealed that SETD2, BAP1 and MTOR especially harbored more mutants in the high-risk group than in the low-risk group (Figure 6A). Additionally, the Wilcoxon rank-sum test suggested that the MAG nomogram risk scores were significantly higher in the high TMB group than in the low TMB group (*P* = 2.875e-05). Moreover, we further analyzed the survival significance of TMB in ccRCC and found that higher TMB levels were associated with an increased risk of progression events with *P* = 0.01 (Figure 6C) and worse OS outcomes with *P* = 0.035 (Figure 6D). We accordingly speculated that ccRCC patients with high MAG nomogram scores suffered from higher TMB levels which was also proven to be a risk factor in ccRCC.

### GSEA

**Figure 7:**
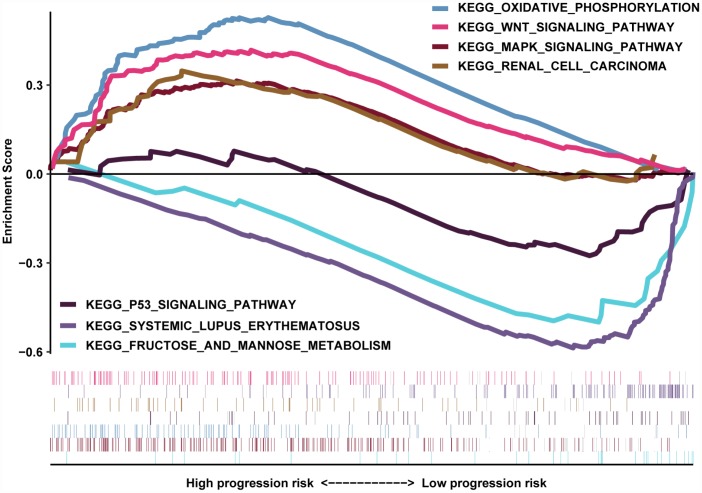
**GSEA results revealed the significantly enriched biological processes between two nomogram-score levels.**

The transcriptome data of 517 ccRCC patients were selected for the gene set enrichment analysis (GSEA) procedure using the MAG nomogram scores as the reference phenotype. We observed that oxidative phosphorylation, the Wnt signaling pathway, the MAPK signaling pathway and renal cell carcinoma crosstalk were upregulated in the high-risk group. However, the P53 signaling pathway, systemic lupus erythematosus and fructose metabolism crosstalk were downregulated in the low-risk group (Figure 7). All of these aberrant pathways were enriched for hallmarks of malignant tumors with a false discovery rate (FDR) of < 0.05.

## DISCUSSION

Malignant progression and a high rate of tumor recurrence have made ccRCC the most lethal type of kidney cancer in the urinary system [18]. Previous studies mainly focused on the screening of biomarkers differentially expressed between tumor and nontumor tissues [19, 20]. However, there is a possibility of missing significant genes when dealing with the bulk transcriptome profiling of cell populations [21, 22]. Moreover, elucidating the underlying mechanisms associated with the metastasis and recurrence of ccRCC is relatively more meaningful. In our study, we analyzed the raw scRNA data of 121 cells with superior quality to depict the genomic features between primary and metastatic ccRCC, during which we identified and confirmed the 17 pivotal MAGs. Furthermore, we utilized internal and independent external cohorts to validate our robust MAG signature. Accordingly, an integrative MAG nomogram model was constructed incorporating four variables to predict cancer-specific tumor progression with high efficiency. Multiomics analysis indicated that high MAG nomogram risk scores correlated with a high TMB, which was demonstrated as a risk factor for prognosis. In another aspect, these findings suggested that the scRNA-seq method combined with validations in cohort populations was proven to be a powerful and sensitive strategy to derive significant gene signatures with potential clinical value in ccRCC.

The scRNA-seq profiling of cells was performed with strict quality control, and we filtered out cells with high proportions of mitochondrial DNA sequencing (> 5%), which was a confounding factor for the statistical results. The subsequent PCA analysis, a method of linear dimensionality reduction, exhibited good discrimination across the three subpopulations of ccRCC cells, indicating the accuracy and reliability of the included data. To thoroughly characterize the high dimensional variables, we finally utilized the t-SNE algorithm to conduct nonlinear dimensionality reduction, and we successfully classified the cells into two categories consisting of primary and metastatic subgroups, in accordance with the actual cell type. The PDX model was constructed to maintain similar pathology and genetic heterogeneity, and there were no significant differences between patient-derived mRCC and PDX-mRCC cell subsets in our cluster analysis. Based on these results, the marker genes were screened between two clusters, and we finally selected the top 44 as the significant signature (hub genes), which was closely associated with metastasis and thus might determine the overall prognosis of ccRCC. Furthermore, we conducted the single-cell trajectory analysis based on RNA-seq using the molecule packaging, which arranges the cells ranked in a simulated chronological order, and illustrated their developmental trajectories, including cell differentiation and other biological processes. In our study, we utilized marker genes with unsupervised learning to mimic the trajectory map. Though the annotations of two clusters were all epithelial cells, the significant curve tendency revealed differential genomic alterations from primary tumors to metastatic tumors.

Some of the 17 identified MAGs have already been reported to play essential roles in tumor progression across malignancies. Bigot P et al. performed genome-wide association studies and identified the RCC risk allele at 12p12.1, a hazard variant in an enhancer that upregulates the expression of BHLHE41, in turn inducing IL-11 to promote tumor growth [23]. BNIP3 acts as a proapoptotic factor, and the identified FoxO-BNIP3 axis plays a unique role in the regulation of mTORC1 and cell survival under energy stress [24]. CCL20 and CXCL1 are chemokines mediated by cancer cells or other immune cells in the tumor microenvironment and are associated with the differentiation and progression of ccRCC [25–27]. Moreover, we also detected a list of genes involved in the energy metabolism pathway consisting of MT-ND3, MT-ND4, MT-RNR2 and MT2A. Previous studies have highlighted the essential roles of these genes in cancer metabolic regulation [28–30]. We observed that the four genes were all upregulated in the metastatic cell cluster and that high expression levels of all these genes correlated with higher probabilities of tumor progression, providing another direction for our subsequent research.

For population validation, we utilized another ICGC cohort as the external data set to further test our MAG signature and found the clinical value of MAGs in predicting OS or PFS. The subsequent multivariate Cox regression analysis excluded the three variables of TNM stages due to incomplete data, conflicting or non-significant results. Given the close correlations of MAGs with metastasis, we still considered whether the factor of M stage could be further integrated into the final nomogram model, and large samples for training are warranted in the future. In addition, we observed the mutation features in two MAG nomogram risk groups and found that SETD2, BAP1 and MTOR revealed more mutated frequencies in the high PFS-risk group. We accordingly speculated that the four tumor-driver mutants might promote the progression of ccRCC and that a high TMB was also proven to be a potential risk factor associated with MAGs. TMB or mutational signatures revealed the process of mutation accumulation in tumors and were demonstrated to be effective predictors of the response to immunotherapy. Whether the MAGs possess potential predictive value for drug therapy remains unclear and would be interesting and valuable to investigate. Additionally, to further prove the validity of the MAGs, we conducted the functional enrichment analysis in several common biological pathways, including oxidative phosphorylation, the Wnt signaling pathway, and the MAPK signaling pathway, which are vital signaling crosstalk pathways in ccRCC [31–33].

Of note, one of the strengths of our work was the combination of scRNA-seq and validation in cohorts, in which we further conducted analyses on the internal and external data set to demonstrate the robustness of the MAG signature that we identified. Compared with traditional bulk transcriptome sequencing analysis in ccRCC [34, 35], scRNA-seq could possess the superiority to find the potential hub markers which might be covered in bulk sequencing. In addition, we integrated multiomics, large-sample analysis to characterize the MAGs involved in the evolution of pRCC to mRCC. Nevertheless, there are still several weaknesses for further optimization. First, the cells or tumor tissues were mostly derived from American or European populations, and whether the identified MAGs were appropriate for those of Asian ethnicity remain indefinite; thus, we should validate our findings in cohorts from local hospitals. Though the signature or nomogram was validated well in large ccRCC populations, supplemental basic experiments are still warranted to uncover the specific mechanisms of MAGs in the promotion of tumor development.

In conclusion, this study is the first to screen marker genes based on scRNA-seq that were validated in a large set of ccRCC samples. We not only depicted the genomic features and heterogeneity between pRCC and mRCC but also found several MAGs, providing a plausible signature for predicting prognosis and underlying evidence for drug discovery against metastasis.

## MATERIALS AND METHODS

### Acquisition of cell samples and ccRCC population cohorts

We obtained the raw data of 121 cell samples with single-cell transcriptome profiling from GSE73121 via the Gene Expression Omnibus (GEO) database (https://www.ncbi.nlm.nih.gov/geo/). The ccRCC tumor cells from parental mRCC, PDX-mRCC and PDX-pRCC were finally analyzed in our study after filtering out poor-quality cells. We then merged the transcriptome data into one matrix and conducted the normalization process using the limma package. We downloaded the expression profiles of 537 ccRCC samples from the TCGA database (https://portal.gdc.cancer.gov/) and of 91 patients from the ICGC database (https://icgc.org/). The normalization of transcriptome count was conducted by the edgeR package (Version 3.26.8). In addition, we also obtained somatic mutation data processed by VarScan software from the “Masked Somatic Mutation” category in TCGA. We utilized the Maftools package (Version 2.0.16) to visualize the genomic alterations for files in Mutation Annotation Format (MAF) [36]. Moreover, we collected data on the complete clinical characteristics of 628 ccRCC samples from two independent cohorts, including age, sex, TNM stage, tumor grade, pathological stage, follow-up time and vital status.

### Processing of single-cell RNA-seq data

We extracted the transcriptome sequencing data of 121 tumor cells isolated from patient-derived mRCC, PDX-mRCC, and paired PDX-pRCC using GRCh38 as the reference genome. We utilized the Seurat package to generate the object and filtered out cells with poor quality [37]. The reading depth of scRNA-seq was 10x genomics based on Illumina HiSeq 2500. Then, we conducted standard data preprocessing, where we calculated the percentage of the gene numbers, cell counts and mitochondria sequencing count. We excluded genes with less than only 3 cells detected and disregarded cells with less than 200 detected gene numbers. The proportion of mitochondria was restricted to less than 5%. Afterwards, we identified the gene symbols with significant differences across cells and constructed a characteristic variance diagram. In addition, we conducted PCA with linear dimensionality reduction and identified the significantly available dimensions of data sets with an estimated P value [38]. Importantly, we further utilized the t-SNE algorithm to conduct the cluster classification analysis across cell samples and screened the marker genes between clusters with logFC =0.5 and adjPval =0.05 as the cutoff criteria [39]. The heatmap of the top significant marker genes was illustrated via ggplot2 package (Version 2.2.1) [40]. Finally, we used the marker genes to annotate the cluster and cell categories based on the SingleR package (Version 0.99.13), and pseudotime analysis of cells was performed via the monocle package (Version 2.12.0), which has been commonly adopted for differential expression analysis, clustering, visualization, and other useful tasks on single-cell expression data [41, 42].

### Identification of MAGs in ccRCC population cohorts

Given the already detected marker genes from the scRNA-seq, we further investigated the significant signature associated with survival across the ccRCC samples. First, we randomly classified the whole TCGA-KIRC cohort into two populations as the training and testing groups. Then, we extracted the transcriptome profiles of the hub marker genes from 265 patients with matched prognostic data in the TCGA training data set. A LASSO regression model using glmnet package was performed to identify the prognostic hub genes from the identified markers genes across scRNA-seq. Afterwards, we illustrated the differential distributions of the hub signatures in two clusters across cell samples using bubble plots and scatter diagrams. Furthermore, the MAG signature was calculated as: MAGs = Ʃ(βi * Expi), where βi, the coefficients, represented the weight of each included gene. In the training data set, we used the ROC curve to assess the predictive value of MAGs in predicting OS, and the difference in survival outcomes was evaluated via Kaplan-Meier analysis with the log-rank test. Accordingly, we further validated our MAG signature in an internal testing data set and an external ICGC cohort. In the whole TCGA-KIRC cohort, we characterized the distributions of death or progression/recurrence endpoint events according to the MAG scores. Moreover, we conducted a correlation analysis between the MAGs and clinical variables consisting of TNM stages, pathological stages and tumor grades. We further analyzed the predictive efficiency of the MAGs in predicting ccRCC progression and conducted survival analysis in the total TCGA-KIRC cohort. Finally, the potential association of the MAGs with TNM stages was validated in the ICGC cohort.

### Development of an individualized prediction model for monitoring progression

We merged the MAG signature with other clinical features in the whole TCGA-KIRC cohort. Univariate and multivariate Cox regression methods were conducted to evaluate the significant clinical variables. After excluding the meaningless variables, we established the integrative MAG nomogram model using a GLM. The ROC plot with the AUC and calibration curve were derived to assess the actual predictive significance of the nomogram based on the rms and pROC packages. Additionally, the survival difference between high- and low-nomogram levels was estimated via Kaplan-Meier analysis.

### Profiles of TMB and correlation analysis

The TMB in ccRCC was defined as: TMB = (total count of variants) / (the whole length of exons). We wrote a Perl script to extract all mutation data from 337 patients in the TCGA-KIRC cohort consisting of deletions, insertions, and substitutions across bases and divided the data into two groups according to the MAG nomogram risk scores. The Maftools package was used to illustrate the respective mutation profiling of the two nomogram risk levels by waterfall plot. Afterwards, the differential mutation frequencies of mutants detected more than 5% were compared using the chi-square test between the two nomogram groups. Moreover, TMB was derived for each patient, and the underlying relationship with MAGs was calculated with Pearson correlation analysis with estimated P values. Of note, we also analyzed the survival significance of TMB with OS and PFS in ccRCC.

### Functional pathway analysis between the two MAG nomogram groups

Since we have already classified the TCGA-KIRC cohort into two groups with high and low MAGs-nomogram score levels, we further conducted GSEA using the nomogram score as the phenotype. With the GSEA software via the Java platform, we derived the “c2.cp.kegg.v6.2.symbols.gmt gene sets” from the MSigDB database (http://software.broadinstitute.org/gsea/msigdb) as the reference set. The enriched signaling pathways with FDR < 0.05 were defined as statistically significant.

### Statistical analysis

LASSO regression, Cox regression analysis and Kaplan-Meier curves with the log-rank test were conducted by the glmnet and survival packages. The GLM was established with the rms package. Student’s t test was used for continuous variables, while categorical variables were compared with the chi-square (χ2) test. The Wilcoxon rank-sum test was utilized to compare ranked data with two categories, and the Kruskal-Wallis test was utilized for comparisons among three or more groups. All statistical analyses were conducted in R studio (Version 3.5.3), and we regarded P < 0.05 as statistically significant.

## Supplementary Material

Supplementary Figures

Supplementary Table 1

Supplementary Table 2

Supplementary Table 3

Supplementary Table 4

Supplementary Table 5
