# Supplementary material for: Development and validation of a metastasis-associated prognostic signature based on single-cell RNA-seq in clear cell renal cell carcinoma
Source: Aging (Albany NY). 2019 Nov 20;11(22):10183–202. doi: 10.18632/aging.102434 (PMC6914399; doi:10.18632/aging.102434)
Supplement: Supplementary Figures [file aging-11-102434-s006..pdf]

SUPPLEMENTARY FIGURES

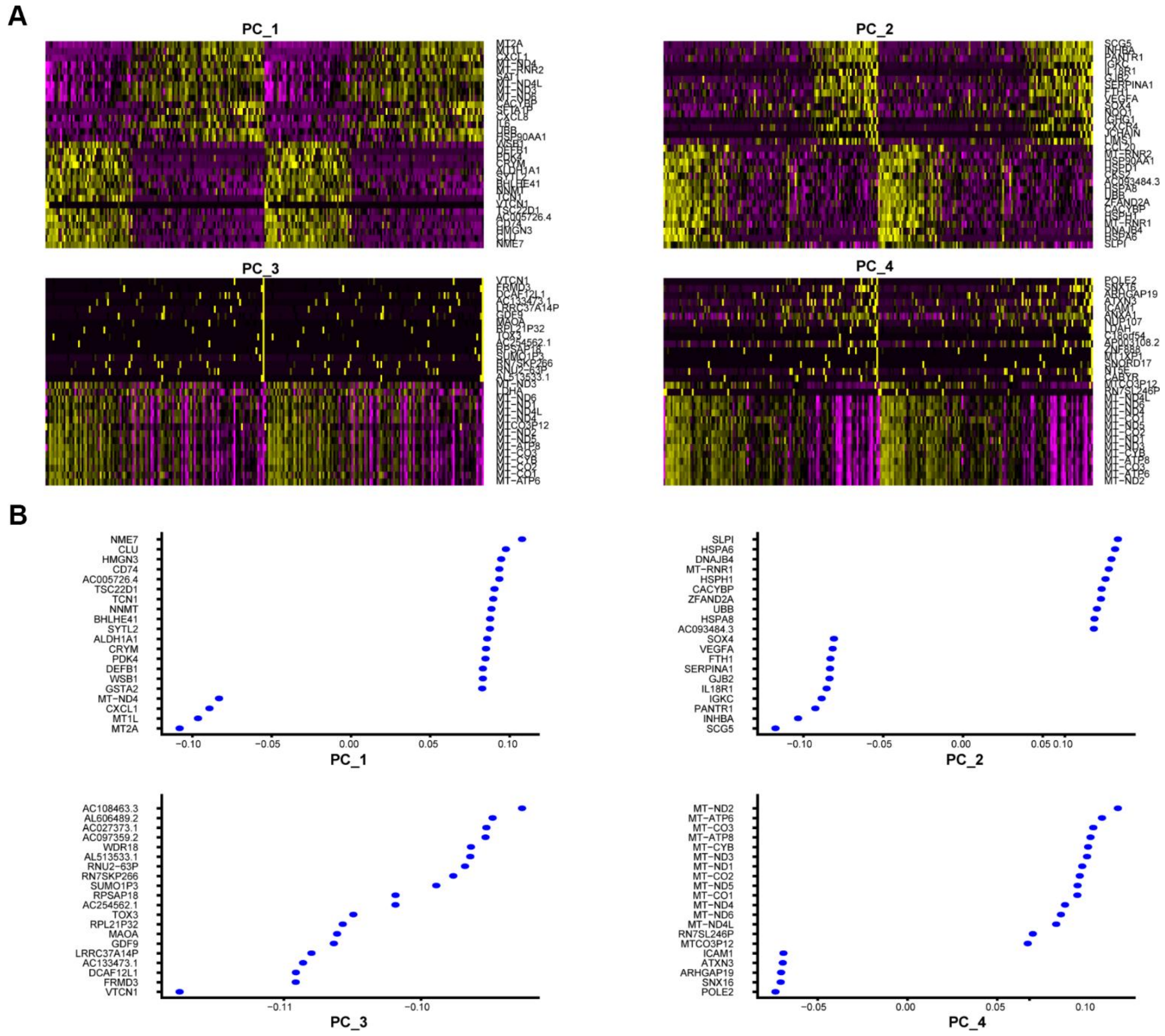

Supplementary Figure 1. The top 4 components from PCA procedure were shown and we exhibited the correlated genes in each component. (**A**) Cluster analysis across each component.The colors ranging from purple to golden yellow represent the expression levels of correlated genes from low to high. (**B**) Correlation analysis of top relative genes in each component.

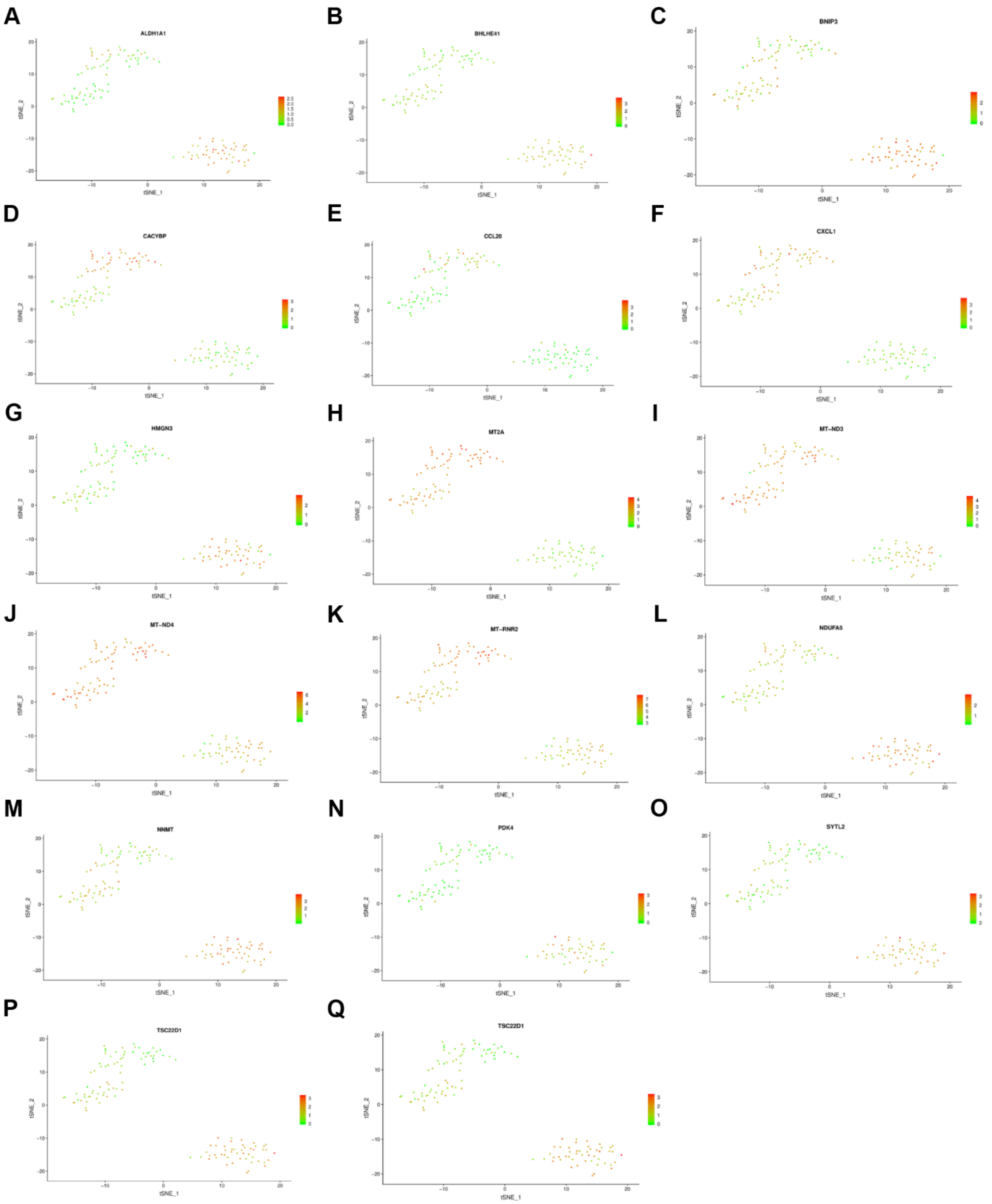

Supplementary Figure 2. The differential expression levels of 17 hub metastasis-associated genes in two clusters from the scRNA-seq.

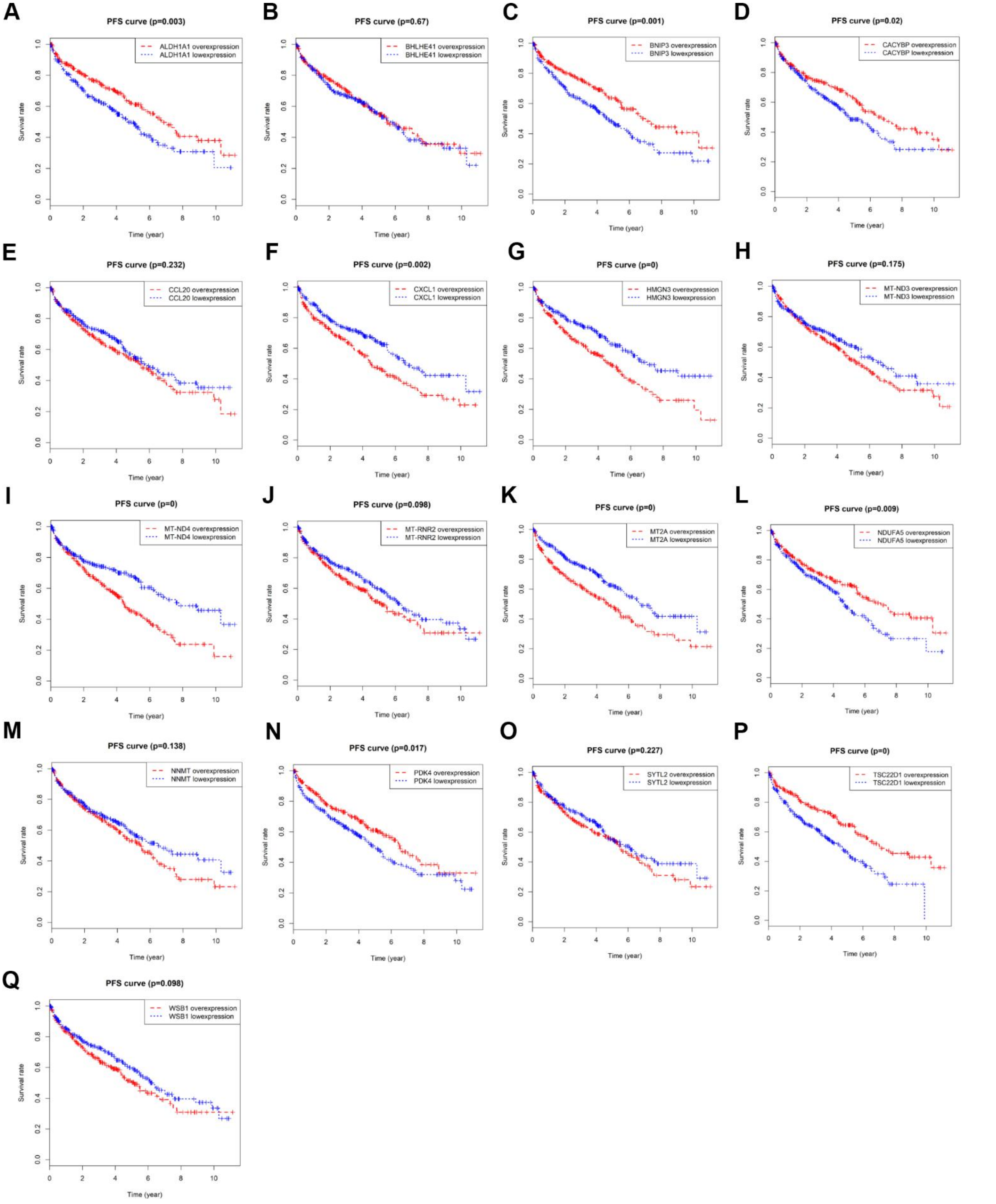

Supplementary Figure 3. Survival analysis of the 17 hub metastasis-associated genes in total TCGA-KIRC cohort, where we observed that most of them correlate with PFS in ccRCC.
